# Assessment of Frontal Sinus Volume and Cranial Base Length Based on Different Vertical Skeletal Patterns: A Cone Beam Computed Tomographic Study

**DOI:** 10.7759/cureus.55099

**Published:** 2024-02-27

**Authors:** Arshya A Kumar, Srirengalakshmi Muthuswamy Pandian

**Affiliations:** 1 Orthodontics and Orthopedics, Saveetha Dental College and Hospitals, Saveetha Institute of Medical and Technical Sciences, Saveetha University, Chennai, IND; 2 Orthodontics and Dentofacial Orthopaedics, Saveetha Dental College and Hospitals, Saveetha Institute of Medical and Technical Sciences, Saveetha University, Chennai, IND

**Keywords:** cranial base length, dental, skeletal malocclusion, cbct, frontal sinus

## Abstract

Introduction

Evaluating craniofacial growth is an essential component of orthodontic treatment, and it is assessed by examining the cranial base. The anterior cranial base is regarded as a stable structure, and the frontal sinus is also recognised as a contributing component in the formation of the cranial base. The frontal sinus, a cavity present in the frontal bone, displays variation in both size and shape and has an impact on the overall structure of the skull and face. This study aims to evaluate the impact of vertical skeletal pattern and gender on the volume of the frontal sinus.

Materials and methodology

In this study, 90 cone beam computed tomography (CBCT) scans from the record’s section were included, comprising 46 males (55.44%) and 44 females (48.88%) aged 20 to 35 undergoing orthodontic treatment. The assessment involved evaluating vertical skeletal patterns using a lateral cephalogram derived from the CBCT scans, and volumetric analysis of the frontal sinus was conducted using Dolphin Imaging software (version 11.9; Dolphin Imaging and Management Solutions, Chatsworth, California). Statistical analysis was performed on the collected data using SPSS software, version 20.0 (IBM Corp., Armonk, NY). Pearson correlation, a one-way ANOVA test to determine any statistically significant differences between the means of both frontal sinus volume and cranial base length groups individually and an independent t-test to compare the sample means between the frontal sinus volume and cranial base length groups were performed.

Results: A non-significant association was observed between frontal sinus volume and cranial base length in skeletal open bite (p = 0.73) and skeletal deep bite (p = 0.12) between males and females, which implies there is no substantial association between frontal sinus volume (p = 0.08) and cranial base length (p = 0.41) in the different vertical skeletal patterns.

Conclusion: Frontal sinus volume was similar in subjects with a skeletal open bite and a deep bite. Males and females did not show a difference in frontal sinus volume. Hence, it was concluded that frontal sinus volume and anterior cranial base cannot be used as parameters to predict vertical malocclusions.

## Introduction

The frontal sinus is a paired, air-filled sinus associated with the nasal cavity. It exhibits a tortuous structure and is of dissimilar shapes and sizes [1]. It is located dorsally to the superciliary arches and the nasal root and is situated adjacent to delicate structures [2]. The frontal sinus has a crucial role in decreasing the weight of the cranium and shielding the brain from injuries. Septations and bone resorption aid in the independent development of the left and right frontal sinuses [3]. The frontal sinus is not formed during birth, and its subsequent growth begins by the first year of life, develops swiftly at the time of puberty, and ceases to grow by 20 years of age [4]. It is seen radiographically by six years of age. The development of the frontal sinus differs based on the individual, age, ethnicity, and disease [5,6].

These challenges of traditional methods can be overcome through the emergence of modern digital orthodontics, which has broadened the scope of innovative approaches within the field of dentistry and incorporates a diverse range of technologies, including workflows, CBCTs, predictive and design software, 3D printers, and AI-guided navigation systems [7-9]. The morphological limits of the paranasal sinuses in humans were first studied in cadavers by obtaining radiographs or by injecting certain substances [10]. The development of 3-dimensional methods of radiographic evaluation has made it easier to obtain a more accurate method to assess the frontal sinus volume in all dimensions as compared to 2-dimensional methods. The development of various software for 3-dimensional assessment and planning has led to a new epoch in digital dentistry [11].

Assessment of craniofacial growth is a vital part of orthodontic treatment and is evaluated using the cranial base, which consists of an anterior and posterior part, which is a reference landmark [12]. The anterior cranial base has been considered to cease its growth prior to other facial skeletal structures, making it one of the most stable landmarks during superimpositions and cephalometric analysis [13]. The vertical relationship of the maxilla and mandible at the level of the incisors can be correlated with skeletal hyperdivergence (open bite) and skeletal hypodivergence (deep bite) [14]. The etiology of these malocclusions can be facial growth pattern, skeletal, dental, due to habitual aspects, or genetic [15,16]. No research has investigated the relationship between cranial base length and frontal sinus volume in various vertical malocclusions. The present study aims to assess and establish a correlation between cranial base length and frontal sinus volume among individuals of Dravidian descent exhibiting varying vertical skeletal malocclusions.

## Materials and methods

A retrospective investigation involved Dravidian individuals, examining 90 CBCT scans from patients aged 20 to 35 seeking orthodontic treatment at the Department of Orthodontics and Dentofacial Orthopedics, Saveetha Dental College and Hospital in Chennai. The full case records of the subjects were analysed and classified into three groups based on the vertical skeletal patterns. Lateral cephalograms were obtained from the CBCT images by an orthodontist using Dolphin Imaging Software, version 11.9 (Dolphin Imaging and Management Solutions, Chatsworth, California). The vertical skeletal pattern was divided based on Burstone cephalometric analysis. Ar-Go (linear), Ar-Go-Gn, and OP-HP angles were measured to confirm anterior open bite malocclusion, deep bite malocclusion, and normal overbite [16]. Good-quality CBCTs were selected for the study. Adult subjects with fully erupted permanent teeth were included, and individuals with paranasal sinus pathologies, facial asymmetries, craniofacial syndromes, and previous orthognathic surgeries were excluded.

**Table 1:** Mean age, standard deviation, and gender of age groups n= number of subjects

Gender	n	%	Age	
			Mean	Standard deviation
Males	46	53.33	26.46	4.17
Females	44	46.66	25.95	3.59
Total	90	100	26.20	5.5

The CBCTs were taken using a Carestream 9600 CBCT scanner (Onex Corporation, Canada) with exposure of 120 KV at 5 mA, acquisition time of 40 seconds, voxel size of 300μm x 300μm x 300μm and FOV (field of view) of 16 x 17 cm by a single operator. Subjects were asked to stand with their head in the natural head position and maximum intercuspation of the teeth.

Measurement of frontal sinus volume

**Figure 1:**
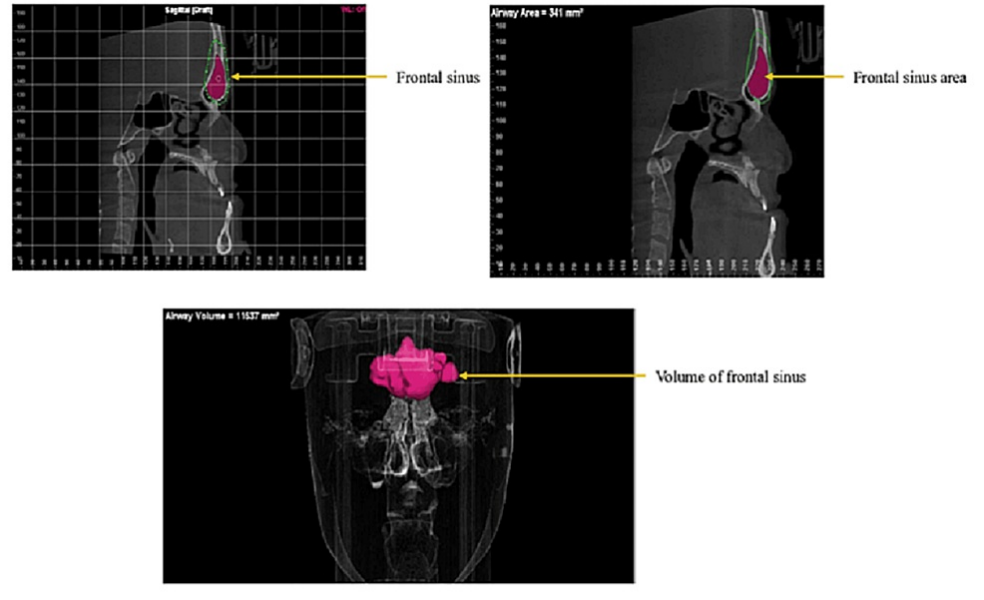
Evaluation of frontal sinus volume using Dolphin imaging software Dolphin imaging software, version 11.9 (Dolphin Imaging and Management Solutions, Chatsworth, California)

Dolphin Imaging software, a semiautomated segmentation tool, was used to evaluate the frontal sinus volume, as shown in Figure 1. The segmentation process involved defining all the walls of the frontal sinus as a region of interest, followed by an evaluation of the frontal sinus volume. Subsequently, the software was utilized to measure the frontal sinus volume in cubic millimetres on the left and right slides. The analysis of the CBCT images and the measurement of the volume were performed by a skilled oral radiologist (AK).

Statistical analysis

The statistical analysis was performed using the SPSS Statistics software, version 20.0 (IBM Corp., Armonk, NY). The intra-examiner agreement was determined by employing the intraclass correlation coefficient (ICC), with the same operator conducting volumetric calculations on 10 CBCTs from each group after 14 days. The significant difference was assessed using the Independent t-test, One-way ANOVA test and Pearson Correlation.

## Results

The inter-rater correlation coefficient exhibits excellent agreement among the examiners for measuring the cranial base length and frontal sinus volume. No substantial association between frontal sinus volume (p = 0.08) and cranial base length (p = 0.41) was noted in the different vertical skeletal patterns, as shown in Tables 2, 3.

**Table 2:** Mean comparison of frontal sinus volume between different vertical skeletal patterns

Volume of Frontal Sinus	N	Mean (mm^3^)	Std. Deviation	One way ANOVA	P value
						Normal Overbite	30	5271.07	2991.72	2.57	0.08
Skeletal Openbite	30	6985.00	3022.14								
Skeletal Deepbite	30	5516.20	3453.33								

**Table 3:** Mean comparison of cranial base length between different vertical skeletal patterns

Cranial base length	N	Mean (mm)	Std. Deviation	One way ANOVA	P value
						Normal Overbite	30	49.89	1.95	0.89	0.41
Skeletal Openbite	30	49.29	1.88								
Skeletal Deepbite	30	49.84	1.97								

Based on the independent t-test results (Table 4), it is concluded that there is no substantial association between the average cranial base length and frontal sinus volume between the genders.

**Table 4:** Subgroup examination of genders between skeletal malocclusion of frontal sinus volume and cranial base length

Groups	Parameter	Gender	Mean	Std. Deviation	Independent test t value	P value
Normal Overbite	FSV	Male	4629.87 mm^3^	2573.68	-1.18	0.30
		Female	5912.27 mm^3^	3321.94		
	CBL	Male	49.65 mm	1.89	0.64	0.37
		Female	50.12 mm	2.06		
Skeletal Openbite	FSV	Male	6490.93 mm^3^	2895.17	0.89	0.34
		Female	7479.07 mm^3^	3164.49		
	CBL	Male	49.42 mm	1.91	0.38	0.97
		Female	49.15 mm	1.91		
Skeletal Deepbite	FSV	Male	5018.87 mm^3^	3004.26	-0.78	0.13
		Female	6013.53 mm^3^	3891.94		
	CBL	Male	50.22 mm	2.01	1.04	0.74
		Female	49.47 mm	1.93		

The correlation between the cranial base length and frontal sinus volume was not significant in different vertical skeletal patterns (Table 5). 

**Table 5:** Pearson correlation between frontal sinus volume and cranial base length in skeletal malocclusions

Groups	Pearson correlation coefficient	P value
Normal Overbite	-0.07	0.73
Skeletal Openbite	0.07	0.73
Skeletal Deepbite	-0.29	0.12

## Discussion

Frontal sinus volume and vertical skeletal pattern

The anterior cranial base, a cranium structure, attains full development ahead of other cranial structures, rendering it a stable entity [17]. The frontal sinus, a noticeable paranasal sinus, develops concurrently with craniofacial structures, offering the potential for early malocclusion prediction [18]. No study has been done previously to assess the cranial base length and frontal sinus volume in different vertical skeletal patterns for their correlation, which could potentially aid in timely diagnosis and treatment planning. The frontal sinus volume measurement was done using Dolphin Imaging software, a semi-automated tool, with malocclusion confirmation based on lateral cephalograms obtained from the CBCT and Burstone cephalometric analysis. The cranial base length and frontal sinus volume showed no correlation in subjects with average overbite (p= 0.73), skeletal deepbite (p = 0.12), and skeletal openbite (p= 0.73). Dhiman et al., in 2015, conducted a study that assessed the various skeletal malocclusions by measuring the frontal sinus and maxillary sinus [19]. AutoCAD software was used to determine the sizes of the two sinuses, and they were correlated with linear and angular cephalometric measurements. The results indicated that individuals with class III malocclusion exhibited a larger frontal sinus area compared to those with skeletal class I and class II malocclusions. A significant difference in the frontal sinus area between males and females was also noted. The average frontal sinus volume reported in recent research was 2.92 ± 2.57 cm^3^. Males showed a larger frontal sinus volume when compared to females, which is 3.74 ± (2.97) and 3.21 ± (2.79) cm^3^, respectively. The frontal sinus volume may differ based on race, age, and gender.

Cranial base length and vertical skeletal pattern

Monirifard et al. performed a study in 2020 to investigate the correlation between anterioposterior cephalometric indicators and cranial base cephalometric indicators within the population of Iran [20]. The findings revealed significantly larger dimensions of the cranial base in men compared to women. Moreover, the study observed that the anterior cranial base length was higher in Class I malocclusion in contrast to Class II malocclusion. Additionally, a smaller cranial base angle was demonstrated in skeletal Class III malocclusion compared to skeletal Class II malocclusion. In 2020, Ahmed et al. investigated the relationship between cranial base morphology and skeletal malocclusion in Sudanese orthodontic patients [21]. The study revealed that there were no significant differences in cranial base angle and cranial base length among the primary malocclusion classes studied. However, there were notable distinctions in jaw size among the three groups, with the Class II group exhibiting a longer maxilla and the Class III group having a longer mandible. The research identified a positive correlation between cranial base lengths and the lengths of the maxilla and mandible across the three groups. Despite this correlation, the study concluded that cranial base angulation and lengths do not play a major role in the development of malocclusion. No research has been conducted to assess the connection between cranial base length and vertical skeletal patterns. The findings of the current study indicate that there is no significant correlation observed between cranial base length and vertical skeletal patterns.

Frontal sinus volume and cranial base length

Nathani et al. in 2016 conducted a study assessing the potential of the frontal sinus as a growth predictor of growth patterns in children [22]. They utilized lateral cephalograms from patients and employed VistaDent software to confirm malocclusion. Additionally, AutoCAD software was utilized for the precise calculation of the frontal sinus area. It was concluded that the mean incremental growth of the frontal sinus varied across different growth patterns. The area of the frontal sinus was noted to be 4.30 ± 1.23 for the vertical growth pattern, 1.86 ± 0.24 for the horizontal growth pattern, and 1.52 ± 0.71 for the average growth pattern. There was a significant difference noted in various growth patterns. The present study evaluated the frontal sinus volume in different vertical skeletal patterns as it is more accurate as compared to the area and concluded that the frontal sinus volume for average overbite was 5.27 ± 2.99, skeletal openbite was 6.98 ± 3.02, and skeletal deepbite was 5.51 ± 3.45. The absence of a substantial correlation between the frontal sinus volume and cranial base length precludes their utility as predictors for vertical malocclusions. This is contrary to the published literature. The 3-dimensional methodology of the current study is more reliable as compared to 2-dimensional methods of evaluation. More evidence and a larger sample size is required to further confirm the correlation. A previous study by Kumar et al. in 2023 assessed the sphenoid sinus volume and cranial base length in various sagittal skeletal patterns. Subjects exhibiting Class II skeletal malocclusion displayed a greater sphenoid sinus volume compared to those with other malocclusions. Among females with Class III skeletal malocclusion, the sphenoid sinus volume exceeded that of males. There was no observed correlation between sphenoid sinus volume and cranial base length [23]. In the present study, frontal sinus volume was assessed in vertical malocclusions and p= 0.08 which indicates no significant difference between the two groups. Said et al. in 2017 assessed the relationship between frontal sinus size and anterior occlusion [24]. They employed photographs of the subjects, lateral cephalograms, and PA radiographs to categorize individuals into malocclusion. No notable distinction was seen between the skeletal class II and III groups. The current study examined the association between frontal sinus volume and cranial base length and determined that there was no statistically significant correlation between the two variables. Metin-Gürsoy et al in 2020 conducted a three-dimensional analysis of the frontal sinus morphology in relation to diverse vertical facial development [25]. Patients' CBCT scans were categorized into hyperdivergent, hypodivergent, and average growth patterns based on the posterior angle sum prior to the orthodontic treatment. Measurements of frontal sinus dimensions were performed on sagittal, coronal, and axial sections of CBCT images. Additionally, lateral cephalograms derived from the same CBCT scans were used for craniofacial assessments. The study revealed no significant disparity in frontal sinus height among the groups. However, in the hyperdivergent group, both the right (0.020) and left (0.041) frontal sinus widths, as well as the anteroposterior frontal sinus dimension (0.017), were notably reduced. The present study assessed 3-dimensional measurements which provides a more accurate result when compared to 2-dimensional measurements.

Limitations

The limitation of the study was that only the Dravidian population was assessed in the current study, whereas prior investigations have proven the variation in the volume of paranasal sinus and cranial base length across diverse racial groups. A more extensive sample size could enhance the result evaluation.

## Conclusions

The predictive capacity of both frontal sinus volume and cranial base length for vertical skeletal malocclusion is constrained. This constraint is evident in several scenarios, such as skeleton open bite, deep bite, and average overbite, and it applies to individuals of both genders. Furthermore, there is no substantial correlation observed between frontal sinus volume and cranial base length, indicating that alterations in one of these variables do not align with alterations in the other. The absence of a robust connection underscores the independence of frontal sinus volume and cranial base length in relation to vertical skeletal issues. Thus, depending exclusively on frontal sinus volume or cranial base length may not provide a comprehensive or reliable foundation for forecasting the existence or intensity of vertical skeletal problems. To have a comprehensive understanding of the intricate elements influencing vertical skeletal relationships in both men and women, it may be necessary to employ a more comprehensive methodology that encompasses a wider array of orthodontic examinations. Additional research could involve increasing the sample size and incorporating a diverse range of ethnicities rather than focusing solely on one ethnic group. The evaluation of frontal sinus volume and cranial base length can be extended to include various sagittal skeletal patterns, such as class I, class II, and class III malocclusion.
